# Advances on Functional Foods with Antioxidant Bioactivity

**DOI:** 10.3390/foods15020341

**Published:** 2026-01-17

**Authors:** Carla S. Carneiro, Igor A. Rodrigues

**Affiliations:** 1 Departamento de Produtos Naturais e Alimentos, Faculdade de Farmácia, Universidade Federal do Rio de Janeiro, Rio de Janeiro 21941-902, RJ, Brazil; 2 Programa de Pós-Graduação em Ciências Farmacêuticas, Faculdade de Farmácia, Universidade Federal do Rio de Janeiro, Rio de Janeiro 21941-902, RJ, Brazil

Oxidative stress (OS), an imbalance between the production of free radicals and the body’s ability to detoxify them, has been linked to a wide range of diseases. Specifically, it is implicated in the onset and/or progression of several neurodegenerative disorders, including Alzheimer’s, Parkinson’s, Huntington’s, and amyotrophic lateral sclerosis [1,2]. In addition, OS is recognized as an important factor contributing to metabolic syndrome (MetS), a group of conditions that include elevated blood pressure, high blood sugar, excess body fat, and dyslipidemia [3]. In contrast, dietary patterns rich in whole grains, fruits, and vegetables, combined with low intake of animal fat, have been shown to attenuate OS in individuals with MetS [4].

The present Special Issue of Foods, Advances on Functional Foods with Antioxidant Bioactivity, brings together a comprehensive and unified set of studies that reflect the evolution of antioxidant research in food. Together, these articles go beyond simple antioxidant measurement. They show how processing, food structure, biological differences, and scientific validation all contribute to determining the true value of antioxidant-rich foods.

A recurring theme throughout this Special Issue is the dual role of food processing as both a potential source of antioxidant degradation and a powerful tool for functional enhancement. Silva et al. (Contribution 1) demonstrated that thermal intensity is a decisive factor in preserving phenolic compounds and anthocyanins in whey–açaí beverages, with excessive heating leading to pronounced antioxidant losses, while milder conditions maintain both functional and sensory quality. In turn, Tangjaidee et al. (Contribution 2) highlighted how innovative processing strategies, including fermentation combined with vacuum impregnation, can significantly enrich coffee matrices with bioactive compounds. Hong et al. (Contribution 3) demonstrated that gamma radiation mutagenesis represents an effective technological approach to enhance seed pigmentation, phenolic accumulation, and antioxidant capacity in colored wheat, while maintaining agronomic performance. These studies reinforce the influence of technological interventions on food antioxidant potential.

The influence of food matrix composition and ingredient functionality is another central contribution of this collection. Tomassi et al. (Contribution 4) demonstrated that tomato-based snack bars enriched with plant proteins not only exhibit enhanced antioxidant capacity but also exert antidiabetic, anti-obesity, and anti-inflammatory effects. These findings emphasize that antioxidant activity should be interpreted within the broader context of multifunctionality, where bioactive compounds interact synergistically with nutrients to modulate metabolic responses.

Several studies also highlight the importance of evaluating antioxidant bioactivity beyond the native food matrix, particularly with respect to bioaccessibility and physiological relevance. Ghion et al. (Contribution 5) showed that simulated gastrointestinal digestion of kombucha results in the release of bioaccessible phenolic compounds while preserving microbial viability, underscoring the need to consider digestion-mediated transformations when assessing food functional potential. The study by Pérez-Beltrán et al. (Contribution 6) on fruit-based mango and pineapple bars integrated in vitro enzymatic assays, in silico modeling, and in vivo glycemic response, and it provided an interesting mechanistic framework that strengthens the biological plausibility of antioxidant-related metabolic effects.

Environmental, genetic, and biological variability emerged as decisive factors shaping antioxidant composition and activity. Lavinas et al. (Contribution 7) demonstrated that seasonal variation affected the chemical and antioxidant profiles of *Melipona mondury* and *Melipona bicolor* honey, emphasizing the ecological dimension of functional food quality. Osei et al. (Contribution 8) demonstrated that sesame seed color is strongly associated with phenolic content and antioxidant capacity, with black varieties consistently exhibiting higher levels of bioactive compounds than lighter-colored seeds. In addition, geographic origin significantly influenced the chemical and functional profiles observed, underscoring the combined effects of genotype and growing conditions. Together, these studies reinforce the need to contextualize antioxidant data within biological and environmental frameworks rather than treating them as fixed attributes.

Importantly, several contributions extended antioxidant research toward health-related outcomes and mechanistic insight. Geng et al. (Contribution 9) demonstrated that green radish polysaccharides mitigate alcohol-induced liver injury through attenuation of oxidative stress, restoration of endogenous antioxidant defenses, and modulation of the gut–liver axis. Likewise, fermentation of legumes with *Aspergillus sojae* revealed that microbial biotransformation can release bound phenolic compounds, thereby enhancing antioxidant and antidiabetic activities (Contribution 10). These studies highlight how antioxidant bioactivity intersects with inflammation, metabolism, and microbiota-mediated pathways.

The Special Issue also embraces a translational perspective, bridging food science and biomedical relevance. The review by Martínez-Zamora (Contribution 11) on hydroxytyrosol synthesizes evidence showing that this olive-derived phenolic compound exerts neuroprotective effects through redox modulation, activation of Nrf2 signaling, and suppression of NF-κB–mediated inflammation. By connecting dietary antioxidants with neurodegenerative disease models, this contribution highlights the expanding horizon of functional foods and nutraceuticals as part of preventive and supportive health strategies.

Taken together, the studies included in this Special Issue collectively advance the field by emphasizing that antioxidant bioactivity cannot be fully understood through isolated chemical assays alone. Instead, meaningful evaluation requires integration of processing effects, matrix interactions, bioaccessibility, biological variability, and mechanistic validation. It is our hope that this Special Issue will not only serve as a reference for current advances but also stimulate future research focused on translational, system-level approaches to functional food development.
